# Comparative Assessment of Lower Urinary Tract Infections in Hospitalized Adults from Western Romania: A Retrospective Cohort with Microbiological Analysis

**DOI:** 10.3390/microorganisms13051130

**Published:** 2025-05-14

**Authors:** Adela Benea, Mirela Turaiche, Ovidiu Rosca, Elena Hogea, Madalina-Ianca Suba, Norberth-Istvan Varga, Uday Shree Akkala Shetty, Daniel Porav-Hodade, Ileana Enatescu, Adrian Cosmin Ilie, Ciprian Rachieru, Daniel-Florin Lighezan, Oana Silvana Sarau, Cristian Andrei Sarau

**Affiliations:** 1 Doctoral School, Faculty of Medicine, “Victor Babes” University of Medicine and Pharmacy Timisoara, 300041 Timisoara, Romania; adela.benea@umft.ro (A.B.); madalina.suba@umft.ro (M.-I.S.); norberth.varga@umft.ro (N.-I.V.); 2 Methodological and Infectious Diseases Research Center, Faculty of Medicine, “Victor Babes” University of Medicine and Pharmacy Timisoara, 300041 Timisoara, Romania; paliu.mirela@umft.ro (M.T.); rosca.ovidiu@umft.ro (O.R.); hogea.elena@umft.ro (E.H.); 3 Internal Medicine, Southern Regional Medical Center, Riverdale, GA 30274, USA; uakkalashetty@phsi.us; 4 Department of Urology, The “George Emil Palade” University of Medicine, Pharmacy, Science, and Technology of Targu Mures, 540139 Targu Mures, Romania; 5 Discipline of Neonatology, Faculty of Medicine, “Victor Babes” University of Medicine and Pharmacy Timisoara, 300041 Timisoara, Romania; 6 Department III Functional Sciences, Division of Public Health and Management, Faculty of Medicine, “Victor Babes” University of Medicine and Pharmacy Timisoara, 300041 Timisoara, Romania; ilie.adrian@umft.ro; 7 Department I of Internal Medicine, Faculty of Medicine, “Victor Babes” University of Medicine and Pharmacy Timisoara, 300041 Timisoara, Romania; ciprian.rachieru@umft.ro (C.R.); dlighezan@umft.ro (D.-F.L.); csarau@umft.ro (C.A.S.); 8 Center for Advanced Research in Cardiovascular Pathology and Hemostaseology, “Victor Babes” University of Medicine and Pharmacy Timisoara, 300041 Timisoara, Romania; 9 Department V of Internal Medicine, Discipline of Hematology, “Victor Babes” University of Medicine and Pharmacy Timisoara, 300041 Timisoara, Romania; oana.sarau@umft.ro

**Keywords:** urinary tract infections, hospitals, catheter-related infections, antibiotic resistance, antibiotic stewardship

## Abstract

Urinary tract infections (UTIs) remain a leading cause of healthcare-associated morbidity, particularly in patients with indwelling urinary catheters. This study aimed to compare catheter-associated (CAUTIs) and non-catheter-associated UTIs of the lower tract among hospitalized adults in Western Romania, identify potential predictors of prolonged hospital stay, and explore the interplay of inflammatory markers and clinical outcomes. We retrospectively examined 130 patients diagnosed with UTIs from 2020 to 2024. Demographic data, comorbidities, laboratory parameters (CRP, procalcitonin, fibrinogen, white blood cell counts), and microbiology results were assessed. Patients were divided into CAUTI (n = 72) and non-catheter UTI (n = 58) groups. CAUTI patients had a significantly longer mean hospital stay (13.9 ± 4.3 vs. 11.7 ± 3.8 days, *p* = 0.01). *E. coli* (29.2%), *Klebsiella pneumoniae* (18.5%), and mixed flora (11.5%) predominated overall, with *Pseudomonas aeruginosa* trending higher in CAUTIs (15.3% vs. 5.2%). Diabetic status correlated with higher CRP (54.7 ± 18.2 vs. 46.9 ± 15.7 mg/dL, *p* = 0.04) and increased intensive care unit (ICU) admission (23.5% vs. 9.4%, *p* = 0.03). In a subgroup of 65 patients, CRP demonstrated a moderate positive correlation with length of stay (r = 0.47, *p* = 0.02). Logistic regression indicated that CAUTI was associated with 2.3-fold higher odds of extended hospitalization (95% CI: 1.2–4.4, *p* = 0.02), adjusting for age, diabetes, and CRP levels. CAUTIs are linked to more resistant pathogens, longer hospitalizations, and potentially greater clinical complications. Diabetes further compounds risk, as reflected in higher inflammatory markers and ICU admissions.

## 1. Introduction

Urinary tract infections (UTIs) remain one of the most prevalent healthcare-associated infections globally, exerting a significant burden on both patients and healthcare systems [1,2,3]. In hospital settings, catheter-associated urinary tract infections (CAUTIs) account for a substantial proportion of nosocomial infections, placing patients at higher risk for bacteremia, increased morbidity, and prolonged hospital stays [4,5]. The World Health Organization (WHO) and other international bodies have repeatedly highlighted the threat of rising antimicrobial resistance among uropathogens, which complicates the treatment landscape [6,7].

In particular, the hospitalized population presents a more complex scenario due to factors such as advanced age, multiple comorbidities, and the frequent use of invasive devices [8,9]. These variables contribute to the selection and spread of multidrug-resistant organisms (MDROs), which not only escalate healthcare costs but also jeopardize patient outcomes [10]. In this context, the appropriate management of UTIs—especially those linked to indwelling catheters—demands a robust understanding of local epidemiology and resistance patterns [11].

Romania, like many countries in Eastern Europe, has experienced an increase in antibiotic-resistant infections over the past decade [12,13]. National and regional data highlight the increasing rates of extended-spectrum beta-lactamase (ESBL)-producing Enterobacterales, methicillin-resistant *Staphylococcus aureus* (MRSA), and other drug-resistant organisms [14,15]. In western Romanian hospitals, the overuse or misuse of antibiotics has been flagged as a key driver of resistance [16,17]. Nevertheless, specific analyses that distinguish between CAUTIs and non-catheter-associated UTIs—especially among adults hospitalized for lower UTIs—remain scarce [18,19].

A thorough examination of epidemiological trends in both CAUTIs and non-catheter UTIs is critical for identifying high-risk patients and understanding the spectrum of etiologic pathogens [2,3]. This approach can also uncover the clinical predictors that place individuals at increased risk of complications or recurrence [9]. Such information is pivotal for guiding targeted interventions—ranging from stewardship initiatives to optimized preventive measures—that ultimately reduce morbidity, mortality, and the overall cost of care [10,20].

Comparing CAUTIs with non-catheter UTIs offers valuable insights into potential differences in pathogen distribution, antimicrobial susceptibility profiles, and patient-centric risk factors [4,5]. For instance, hospitalized patients with long-term catheter use may harbor more resistant flora, whereas community-onset UTIs can still be heavily influenced by local antibiotic prescribing practices [13]. Establishing these distinctions helps refine empirical treatment algorithms, ensuring that initial antibiotic choices are both judicious and effective in combating the most likely pathogens [1,17]. Such tailored approaches are especially important in regions grappling with antibiotic overuse and high rates of MDROs, where evidence-based protocols can make a tangible difference in patient care and outcomes [18,19].

Recurrent UTI was defined by IDSA thresholds: ≥2 symptomatic UTIs within 6 months OR ≥3 within 12 months [20,21]. Lower UTI was categorized as infection confined to the bladder or urethra, typically characterized by localized urinary symptoms without flank pain or systemic manifestations, whereas upper UTI (pyelonephritis) encompassed infections involving the renal parenchyma or renal pelvis, often presenting with flank tenderness and systemic features [22,23]. Uncomplicated UTIs were those occurring in non-pregnant patients with normal genitourinary anatomy [1,4]. By contrast, complicated UTIs included cases associated with immunocompromised states, urinary catheters, or underlying structural abnormalities, reflecting higher risks of adverse outcomes and therapeutic failure. Similarly, all male UTIs are by definition complicated [21,24].

The present study provides an in-depth analysis of hospitalized adult patients diagnosed with lower UTIs in a tertiary care hospital in Western Romania. By juxtaposing CAUTIs with non-catheter UTIs, the study aims to delineate the prevailing clinical and microbiological characteristics, identify significant outcome predictors for prolonged hospital stay, and observe the differences between men and women.

## 2. Materials and Methods

### 2.1. Study Design, Setting, and Ethical Considerations

This was a retrospective, observational study conducted at the Victor Babeș Hospital for Infectious Diseases, affiliated with the Victor Babeș University in Timișoara, Romania. Patient recruitment and data collection took place between November 2023 and November 2024, focusing on hospitalized adults (≥18 years) diagnosed with a urinary tract infection (UTI). The hospital functions as a tertiary referral center, providing specialized care for a broad range of infectious diseases across Western Romania. Ethical approval was granted by the institutional review board of Victor Babeș University in Timișoara (Approval No. 50 from 2 October 2023). Informed consent requirements were waived due to the retrospective nature of the study, but patient confidentiality was rigorously maintained in accordance with the Declaration of Helsinki and applicable national regulations. All data were de-identified prior to analysis to safeguard patient privacy.

### 2.2. Sample Size Calculation and Patient Selection

Sample size determination. Regional antimicrobial-resistance surveillance for western Romania (2020–2024) shows that ≈ 20 % of non-catheter lower UTI isolates are multidrug-resistant, whereas ward-level audits of catheterized patients in the same hospitals report resistance proportions approaching 40–45%. To detect an absolute 24-percentage-point difference (20% vs. 44%) in resistance prevalence between non-catheter UTIs (p_1_ = 0.20) and CAUTIs (p_2_ = 0.44) with 80% power and a two-sided α = 0.05, we used the Two-Proportion Superiority module of the ClinCalc Sample Size Calculator, version 2025 (Clinical Calculators LLC). The calculator returned 58 participants per group (N = 116). Allowing a 10% margin for incomplete or misclassified records increased the target to 128 patients.

Our retrospective census of all eligible admissions from January 2020 to December 2024 yielded 130 analyzable cases (72 CAUTI, 58 non-catheter), thereby exceeding the adjusted requirement and preserving the planned 80% statistical power without prolonging the study period. Unequal group sizes are inherent to the observational design but do not materially affect power for the primary comparison.

A total of 156 individuals were identified, of whom 130 met the final inclusion criteria: age ≥18 years, clear documentation of UTI symptoms (such as dysuria, suprapubic or flank pain, fever), and a positive urine culture defined as ≥10^5^ CFU/mL for each organism or ≥10^4^ CFU/mL in symptomatic patients with one or more pathogens [21]. Individuals were excluded if they had incomplete microbiological data, had been transferred from another facility without comprehensive medical records, or were pregnant/postpartum (≤6 weeks). Patients were then divided into two groups. The CAUTI group comprised those with an indwelling urinary catheter in place for at least 48 h before UTI onset [22], whereas the non-catheter group included those who had never had a urinary catheter during the current admission or whose catheter had been removed more than 48 h before symptom onset.

### 2.3. Data Collection

Relevant demographic, clinical, and laboratory variables were retrieved from the hospital’s electronic database and checked for completeness. Extracted variables included age, sex, comorbidities (notably diabetes mellitus and chronic kidney disease), recent or current antibiotic therapy, inflammatory markers (C-reactive protein [CRP], procalcitonin, fibrinogen), white blood cell counts, and absolute neutrophil counts. Microbiological data involved the identification of bacterial species from urine cultures and their respective antibiotic susceptibility profiles. Length of hospital stay was calculated from admission until either discharge or in-hospital death, and an extended stay was defined as more than 12 days in the hospital.

Culture data were obtained from the laboratory information system that included the pathogen and standard resistances in accordance with laboratory SOPs. Bacterial identification and antibiotic susceptibility testing were conducted following standard protocols established by the Clinical and Laboratory Standards Institute (CLSI) [25]. Antimicrobial-susceptibility testing. All isolates were tested by Kirby–Bauer disk diffusion and, where indicated, Vitek 2 broth microdilution; interpretive breakpoints followed CLSI M100-Ed31 (2021) guidelines. Reference strains *E. coli* ATCC 25922, *P. aeruginosa* ATCC 27853, and *E. faecalis* ATCC 29212 were run in parallel for quality control. Antibiotics judged intrinsically inactive for a given organism (e.g., *cephalosporins* vs. *Enterococcus*) were reported as NA and excluded from resistance tallies.

### 2.4. Statistical Analysis

Data were compiled and analyzed using IBM SPSS Statistics (Version 26.0). Continuous variables were tested for normality with the Shapiro–Wilk test. Those following a normal distribution were summarized as means ± standard deviations and compared using independent-sample *t*-tests. Non-normally distributed variables were presented as medians and interquartile ranges and analyzed with the Mann–Whitney U test. Categorical variables were expressed as frequencies or percentages and compared using chi-squared or Fisher’s exact tests. Spearman’s or Pearson’s correlation coefficients were used to evaluate associations among continuous variables, with particular attention to relationships between inflammatory markers (CRP, procalcitonin, fibrinogen) and length of hospital stay. A multivariate logistic regression model was constructed to identify factors associated with prolonged hospitalization (>12 days). Variables included in the model were those emerging from univariate analyses with a *p*-value < 0.10 and clinical relevance (e.g., CAUTI, diabetes, CRP). Statistical significance was set at *p* < 0.05, and all analyses were two-tailed.

## 3. Results

Table 1 compares the key baseline features of 72 patients with catheter-associated UTIs (CAUTIs) and 58 with non-catheter UTIs. The average age was older in the CAUTI group (62.7 years) than in the non-catheter group (59.3 years). Males constituted a slightly larger proportion in the CAUTI cohort (55.6% vs. 50.0%). Common comorbidities included diabetes, chronic kidney disease, and cardiovascular disease. Although diabetes (30.6% vs. 20.7%) and cardiovascular disease (48.6% vs. 44.8%) were somewhat more frequent in CAUTI patients, neither difference was significant. The most striking result was the significantly prolonged hospital stay among CAUTI patients (13.9 vs. 11.7 days).

*E. coli* remained the most prevalent pathogen overall, appearing in 23.6% of CAUTI isolates and 36.2% of non-catheter isolates, although the difference was not significant. Among CAUTI cases, *Klebsiella pneumoniae* was found in 22.2%, compared to 13.8% in the non-catheter group (*p* = 0.218). Notably, *Pseudomonas aeruginosa* exhibited a higher occurrence in the CAUTI subset (15.3% vs. 5.2%). Mixed flora (polymicrobial infections) appeared in roughly 10–12% of isolates in both groups, whereas *Enterococcus* spp. frequencies remained relatively similar at about 7–8%. The category labeled “Other” (e.g., *Proteus* spp., *Acinetobacter* spp., and other less common organisms) was somewhat more common among non-catheter UTIs (27.6% vs. 18.1%), as seen in Table 2.

Table 3 highlights the proportion of isolates resistant to specific antibiotic classes in CAUTI vs. non-catheter UTI patients. Among CAUTI isolates, 38.9% showed resistance to penicillins, compared to 27.6% in the non-catheter group. Resistance to cephalosporins likewise appeared somewhat higher in CAUTI cases (33.3% vs. 24.1%). Although these differences did not reach statistical significance, the trend is clinically concerning, suggesting that catheters may foster infections by more resistant organisms. Carbapenem resistance remained relatively low (9.7% CAUTI vs. 6.9% non-catheter), which is somewhat reassuring but still noteworthy given carbapenems are often last-resort agents. Fluoroquinolone resistance was 26.4% in the CAUTI group vs. 15.5% in non-catheter patients, again illustrating a tendency for CAUTIs to involve more challenging pathogens.

C-reactive protein (CRP) levels were slightly higher in the CAUTI group (50.8 ± 17.2 mg/dL) than in non-catheter patients (45.1 ± 14.6 mg/dL), reaching borderline significance. Procalcitonin, another biomarker often used to gauge bacterial infection severity, averaged 0.21 ± 0.09 ng/mL in CAUTI vs. 0.17 ± 0.07 ng/mL in non-catheter UTIs, hinting at a possible trend but not meeting the significance threshold. Fibrinogen levels were modestly but significantly higher in CAUTI patients (4.0 ± 0.7 vs. 3.7 ± 0.6 g/L. The white blood cell (WBC) count and absolute neutrophil count were also higher in the CAUTI group, as presented in Table 4.

In Table 5, a multivariate logistic regression model was constructed to identify independent predictors of prolonged hospital stay, defined as >12 days. The presence of a catheter-associated UTI was significantly linked to extended hospitalization (adjusted OR 2.3), implying that CAUTI patients are more than twice as likely to surpass 12 inpatient days compared to their non-catheter counterparts. Age (per 10-year increment) trended toward increased risk (OR 1.2) but did not achieve statistical significance. Diabetes was borderline significant (OR 1.9), reflecting the elevated morbidity in patients with metabolic dysregulation. Notably, CRP demonstrated a significant association (OR 1.2), suggesting that higher inflammatory burden correlates with longer stays.

Notably, LOS correlated positively with CRP (ρ = 0.47, *p* < 0.05), indicating that patients presenting with higher inflammatory responses tended to remain hospitalized longer. Procalcitonin (ρ = 0.40, *p* < 0.05) and fibrinogen (ρ = 0.38, *p* < 0.05) also showed moderate positive correlations with LOS, albeit slightly weaker than CRP’s association. CRP further correlated significantly with procalcitonin (ρ = 0.36, *p* < 0.05) and fibrinogen (ρ = 0.41, *p* < 0.05), consistent with the idea that these markers often rise in tandem during acute infections. Interestingly, age did not show a robust correlation with LOS (ρ = 0.22, *p* ≥ 0.05), nor did it strongly correlate with any single inflammatory marker (Table 6).

Of the 130 patients, 69 (53.1%) were male and 61 (46.9%) were female. Rates of CAUTI were somewhat comparable (58.0% in males vs. 52.5% in females). Hospital stay length also showed no statistically significant difference (mean 13.0 ± 4.0 vs. 12.0 ± 4.1 days). Microbiologically, *Pseudomonas aeruginosa* emerged in 14.5% of males vs. 6.6% of females, hinting that male patients might have a mildly higher risk for this opportunistic pathogen, though it did not reach significance. Carbapenem resistance appeared in about 10.1% of male isolates vs. 6.6% of female isolates. ICU admissions followed a similar non-significant pattern, affecting 15.9% of males vs. 9.8% of females. In contrast, recurrent UTIs (≥2 episodes in the last 6 months) were significantly more frequent in females (32.8%) compared to males (13.0%), as presented in Table 7.

## 4. Discussion

Our findings confirm that in our tertiary-care setting, CAUTI is clinically and microbiologically distinct from non-catheter lower UTI: UTIs in catheterized patients were mostly hospital-acquired, involved a higher proportion of *P. aeruginosa* and ESBL-producing *K. pneumoniae*, and were associated with a median hospital stay two days longer—patterns concordant with large surveillance cohorts that attribute catheterization and biofilm formation to increased exposure to water-borne or device-adapted pathogens and prolonged recovery [4,5]. However, unlike the 30–50% carbapenem resistance reported in Romanian ICUs [26], our carbapenem non-susceptibility remained below 20%, reflecting the fact that our ward rarely uses carbapenems empirically; this discrepancy underlines the value of local stewardship. Diabetes independently doubled the odds of a >12-day admission and was associated with higher CRP, aligning with meta-analyses linking hyperglycemia to impaired neutrophil function and delayed infection resolution [9]; conversely, age lost significance after multivariable adjustment, suggesting that metabolic status, not chronological age, drives prolonged recovery in this cohort. Inflammatory markers (CRP, procalcitonin, fibrinogen) correlated with length of stay, supporting their proposed role as bedside prognosticators [10], while our gender subgroup showed no difference in stay or resistance, mirroring studies where sex effects disappear once device exposure is accounted for. Overall, our data reinforce international guidance that limiting catheter use, rigorously managing diabetic patients, and basing empiric therapy on local susceptibility patterns are pivotal to shortening admissions and curbing resistance in hospitals with moderate but rising MDRO pressure.

Similarly, relevant to the Eastern Europe region, in the studies conducted by Przydacz et al. [27,28], the researchers extensively analyzed treatment patterns for lower urinary tract symptoms (LUTS) and overactive bladder (OAB) within a nationally representative sample of 6005 Polish adults. Their findings underscore a significant discrepancy in healthcare engagement, with only approximately 33% of those experiencing LUTS or OAB actively seeking treatment, and 24–26.4% actually receiving it. Notably, men were more proactive in both seeking (33.5%) and receiving treatment compared to women, which reflects a gender disparity in healthcare utilization. Prescription drugs emerged as the most common intervention, utilized significantly more than other treatment strategies such as lifestyle changes or physiotherapy, which were disappointingly underused. Moreover, while storage and voiding symptoms significantly influenced both men and women to seek treatment, the reception of treatment correlated with voiding symptoms in men and storage symptoms in women. Most of the respondents who received treatment reported satisfaction; however, treatment dissatisfaction was primarily associated with the presence of storage symptoms across genders. Alarmingly, only about 50% of the participants continued with their prescribed treatment plans, indicating a high discontinuation rate, with women being statistically more likely to discontinue treatment than men.

In examining the impact and evolution of antibiotic resistance in urinary tract infections (UTIs) among females, the studies by Mareș et al. [29,30] provide valuable insights into the persistence and shifts in pathogen susceptibility over time. The first study, a cross-sectional retrospective review, reported on 524 female patients with positive cultures for UTIs, revealing a high prevalence of *Escherichia coli*, which accounted for 290 cases (55.34%). This pathogen showed significant resistance to commonly used antibiotics, with resistance rates being 33.1% for amoxicillin–clavulanic acid, 32.41% for trimethoprim–sulfamethoxazole, and 32.06% for levofloxacin, while showing high susceptibility to fosfomycin (96.55%) and imipenem (93.1%). The second study, a sequential multi-year analysis, also focused on *Escherichia coli* among other pathogens and noted a disturbing upward trend in resistance, particularly to levofloxacin, which increased from 28.45% in 2018 to 35.0% in 2022.

In a similar manner, the studies conducted by Chibelean et al. [31] and Petca et al. [32] focused on understanding the antimicrobial resistance (AMR) patterns of uropathogens in Romanian populations, segregated by gender. Chibelean et al. [31] analyzed urine samples from 7719 male patients, identifying *Escherichia coli* as the predominant pathogen with significant resistance to amoxicillin–clavulanic acid (28.03%) and levofloxacin (37.69%), yet displaying high sensitivity to amikacin (91.72%), meropenem (97.17%), and fosfomycin (86.60%). Conversely, Petca et al. [32] examined 13,081 urine samples from female patients, with 1588 meeting their study criteria, revealing similar high resistance rates in *Escherichia coli* to levofloxacin (29.66%) and relatively lower resistance to amoxicillin–clavulanic acid (14.13%). Both studies highlighted considerable resistance to commonly used antibiotics, demonstrating the need for updated and localized resistance data to guide empirical antibiotic therapy. These studies not only align in their findings of *E. coli* as a chief concern but also emphasize the gender-specific nuances of UTI management, suggesting that while the pathogens and resistance patterns may be similar, the scale and impact can vary significantly between male and female populations, necessitating tailored approaches in clinical practice.

In their comprehensive analyses, Borcan et al. [33] and Hogea et al. [34] meticulously quantified the evolving landscape of antibiotic resistance among uropathogens in Romania, each under different circumstances. Borcan et al.’s five-year study at the National Institute of Infectious Diseases revealed escalating resistance among Gram-negative rods, with *Escherichia coli* showing resistance rates of 33.30% to amoxicillin–clavulanic acid and 37.69% to levofloxacin, while exhibiting high sensitivity to fosfomycin (86.60%) and meropenem (97.17%) [33]. The study further highlighted that 48% of Klebsiella spp. and 37% of *Pseudomonas aeruginosa* strains were multidrug-resistant, with all *Acinetobacter baumannii isolates* classified as MDR by the study’s conclusion. Additionally, 37% of *Enterococcus faecium* strains were found to be vancomycin-resistant.

Conversely, Hogea et al. [34], studying the antibiotic resistance trends amid the COVID-19 pandemic, analyzed 378 positive urine samples from 2472 collected, noting that *Escherichia coli*, constituting 46.3% of isolates, had resistance rates that rose from 45.4% in 2020 to 53.8% in 2022 for amoxicillin–clavulanic acid and from 27.5% in 2020 to 47.2% in 2022 for trimethoprim–sulfamethoxazole. *Klebsiella pneumoniae*, accounting for 20.6% of the cases, showed a high level of carbapenemase production at 52.5% and ESBL presence in 24.3% of the strains.

These findings underscore the need for proactive interventions: limiting catheter placement to essential indications, enforcing strict aseptic insertion and maintenance, and adopting vigilant antibiotic stewardship to curb resistance. Special attention should be directed toward diabetic patients, who may require tighter glycemic control and intensified surveillance to mitigate complications. While our results underscore key risk factors and outcome predictors, larger-scale prospective studies with detailed severity-of-illness measures and long-term follow-up are warranted. Such endeavors will refine UTI management protocols, ultimately improving patient care quality, reducing hospital burden, and informing robust infection control strategies in Western Romania and beyond.

This study has several limitations that may affect the interpretation and generalizability of the findings. First, it represents a single-center, retrospective design, which limits the generalizability of the findings to other regions or hospital systems. Second, the relatively modest sample size of 130 patients may limit statistical power, particularly for subgroup analyses and resistance patterns. Third, the accuracy of data extracted from electronic health records depends on thorough medical documentation; incomplete or inaccurate records could lead to misclassification. Fourth, while we used standard definitions for CAUTI and included relevant comorbidities, we did not capture detailed severity-of-illness scores or patient functional status, which could influence outcomes. Fifth, antibiotic prescription practices and timing of cultures may vary by clinician, potentially biasing microbiological results. Lastly, our focus on in-hospital outcomes does not illuminate long-term repercussions, such as recurrent infections post-discharge or readmission rates. Prospective, multicenter research could address these gaps more robustly.

## 5. Conclusions

In this study, we identified significant distinctions between catheter-associated and non-catheter lower UTIs, with CAUTI linked to higher inflammatory markers, more resistant pathogens, and 2.3-fold increased odds of prolonged hospitalization. Diabetes additionally emerged as a notable contributor to adverse outcomes, correlating with elevated CRP levels, more ICU admissions, and longer inpatient stays. Correlation analyses suggested that biomarkers such as CRP, procalcitonin, and fibrinogen moderately predicted extended length of stay, underlining the importance of monitoring the acute inflammatory response.

## Figures and Tables

**Table 1:** Baseline characteristics of CAUTI vs. non-catheter UTI patients.

Characteristic	CAUTI (n = 72)	Non-Catheter UTI (n = 58)	*p*-Value
Age (years), mean ± SD	62.7 ± 14.5	59.3 ± 13.6	0.171
Male, n (%)	40 (55.6)	29 (50.0)	0.528
Diabetes, n (%)	22 (30.6)	12 (20.7)	0.203
Chronic kidney disease, n (%)	9 (12.5)	8 (13.8)	0.828
Cardiovascular disease, n (%)	35 (48.6)	26 (44.8)	0.667
Hospital-acquired UTI ^†^	63 (87.5)	20 (34.5)	<0.001
Total in-hospital days, mean ± SD	13.9 ± 4.3	11.7 ± 3.8	0.003

^†^ Defined as symptomatic UTI onset ≥48 h after hospital admission.

**Table 2:** Pathogen distribution by UTI type.

Pathogen	CAUTI (n = 72), n (%)	Non-Catheter UTI (n = 58), n (%)	*p*-Value
*E. coli*	17 (23.6)	21 (36.2)	0.117
*Klebsiella pneumoniae*	16 (22.2)	8 (13.8)	0.218
*Pseudomonas aeruginosa*	11 (15.3)	3 (5.2)	0.065
Mixed flora	9 (12.5)	6 (10.3)	0.702
*Enterococcus* spp.	6 (8.3)	4 (6.9)	0.760
Other (Proteus, etc.)	13 (18.1)	16 (27.6)	0.194

**Table 3:** Antibiotic resistance patterns in CAUTI vs. non-catheter UTI.

Antibiotic Class	CAUTI (n = 72), n (%) Resistant	Non-Catheter (n = 58), n (%) Resistant	*p*-Value
Penicillins	28 (38.9)	16 (27.6)	0.176
Cephalosporins	24 (33.3)	14 (24.1)	0.251
Carbapenems	7 (9.7)	4 (6.9)	0.565
Fluoroquinolones	19 (26.4)	9 (15.5)	0.134
Aminoglycosides	14 (19.4)	8 (13.8)	0.393
Trimethoprim–Sulfamethoxazole	22 (30.6)	15 (25.9)	0.556
*Escherichia coli*	n = 17	n = 21	
Ampicillin	9 (52.9)	7 (33.3)	0.324
Ceftriaxone (3G CEPH)	6 (35.3)	3 (14.3)	0.249
Ciprofloxacin	5 (29.4)	4 (19.0)	0.703
TMP-SMX	7 (41.2)	6 (28.6)	0.502
Gentamicin	4 (23.5)	3 (14.3)	0.678
Imipenem	0 (0)	0 (0)	ND
*Klebsiella pneumoniae*	n = 16	n = 8	
Ampicillin–Sulbactam	10 (62.5)	3 (37.5)	0.39
Ceftriaxone	9 (56.3)	2 (25.0)	0.211
Ciprofloxacin	7 (43.8)	2 (25.0)	0.657
Meropenem	3 (18.8)	0 (0)	0.526
Gentamicin	5 (31.3)	1 (12.5)	0.621
*Pseudomonas aeruginosa*	n = 11	n = 3	
Piperacillin–Tazobactam	4 (36.4)	0 (0)	0.505
Ceftazidime	3 (27.3)	0 (0)	1
Ciprofloxacin	3 (27.3)	0 (0)	1
Meropenem	2 (18.2)	0 (0)	1
Gentamicin	2 (18.2)	0 (0)	1
*Enterococcus* spp.	n = 6	n = 4	
Ampicillin	2 (33.3)	1 (25.0)	1
Vancomycin	1 (16.7)	0 (0)	1
Linezolid	0 (0)	0 (0)	N/A
Cephalosporins	N/A	N/A	–
Aminoglycosides (high-level)	N/A	N/A	–

**Table 4:** Comparison of key laboratory markers between CAUTI and non-catheter UTI.

Laboratory Marker	CAUTI (n = 72), Mean ± SD	Non-Catheter UTI (n = 58), Mean ± SD	*p*-Value
CRP (mg/dL)	50.8 ± 17.2	45.1 ± 14.6	0.043
Procalcitonin (ng/mL)	0.21 ± 0.09	0.17 ± 0.07	0.005
Fibrinogen (g/L)	4.0 ± 0.7	3.7 ± 0.6	0.010
WBC count (×10^3^/µL)	11.9 ± 3.2	10.8 ± 3.1	0.050
Neutrophils (×10^3^/µL)	8.2 ± 2.8	7.3 ± 2.5	0.055

**Table 5:** Multivariate logistic regression for prolonged hospital stay (>12 days).

Variable	Unadjusted OR	95% CI	*p*	Adjusted OR	95% CI	*p*
CAUTI (yes vs. no)	2.67	1.46–4.88	0.001	2.3	1.22–4.35	0.02
Age (per 10-y↑)	1.28	1.00–1.63	0.048	1.22	0.91–1.63	0.14
Diabetes (yes vs. no)	2.05	1.07–3.92	0.031	1.91	1.00–3.64	0.05
CRP (per 10 mg/dL↑)	1.24	1.05–1.46	0.011	1.2	1.01–1.44	0.04

Model statistics: R^2^ = 0.289; likelihood-ratio χ^2^ (4 df) = 28.7, *p* < 0.001. Hosmer–Lemeshow goodness-of-fit: χ^2^ (8 df) = 5.53, *p* = 0.702. Selection procedure: All variables with univariate *p* < 0.10 (shown above) were entered into the multivariate logistic model by forced entry. The pseudo-R^2^ indicates that the final model explains ≈29% of the variability in prolonged length of stay, and the non-significant Hosmer–Lemeshow test suggests acceptable calibration.

**Table 6:** Correlation matrix among key variables (Spearman’s ρ).

Variable	LOS (Days)	CRP	Procalcitonin	Fibrinogen	Age
LOS (days)	1	0.47 *	0.40 *	0.38 *	0.22
CRP	0.47 *	1	0.36 *	0.41 *	0.25
Procalcitonin	0.40 *	0.36 *	1	0.29	0.16
Fibrinogen	0.38 *	0.41 *	0.29	1	0.19
Age	0.22	0.25	0.16	0.19	1

* Correlation is significant at *p* < 0.05.

**Table 7:** Gender-based subgroup analysis: clinical and microbiological outcomes.

Outcome	Males (n = 69)	Females (n = 61)	*p*-Value
CAUTI, n (%)	40 (58.0)	32 (52.5)	0.528
Mean Hospital Stay (days), mean ± SD	13.0 ± 4.0	12.0 ± 4.1	0.1
*Pseudomonas aeruginosa* n (%)	10 (14.5)	4 (6.6)	0.15
Any Carbapenem Resistance, n (%)	7 (10.1)	4 (6.6)	0.46
ICU Admission, n (%)	11 (15.9)	6 (9.8)	0.31
Recurrent UTIs in Past 6 Months, n (%)	9 (13.0)	20 (32.8)	0.01

## Data Availability

Data availability is subject to hospital approval.
